# Why care resists coding: the moral limits of medical AI

**DOI:** 10.1186/s12910-026-01467-7

**Published:** 2026-05-06

**Authors:** Nils-Frederic Wagner

**Affiliations:** Institute for History, Theory and Ethics of Medicine, University Medical Center Mainz, Mainz, Germany

**Keywords:** Artificial Intelligence in Healthcare, Embodied Moral Agency, Care Ethics, Relational Care, Human Touch

## Abstract

The growing integration of artificial intelligence (AI) into healthcare raises substantial normative questions about the nature of caregiving. While medical AI can improve clinical efficiency and assist with administrative tasks, I argue that patient-facing medical AI cannot straightforwardly substitute for genuine caregiving—understood as an embodied, relational, and morally responsive practice that remains answerable to patients’ vulnerability over time. Drawing on conceptual analysis and empirical evidence, I contend that caregiving exceeds technical competence, requiring context-sensitive responsiveness and a form of moral commitment that grounds responsibility when interpretation and response go wrong. Empirical studies on touch in clinical settings underscore how embodied reassurance and trust are mediated by consent, context, and relational meaning in ways current technological proxies only partially reproduce. Although medical AI may simulate elements of empathy through affective cues and conversational performance, such simulations risk generating interactions that appear supportive while obscuring the locus of responsibility within the care relationship. Hybrid AI-human care models and ‘ethics-by-design’ approaches, while often proposed as solutions, can diffuse accountability and fragment moral responsibility unless governance and clinical workflow design preserve clear human answerability. More critically, increasing reliance on patient-facing medical AI may not only yield inadequate simulations of care, but also contribute to a gradual redefinition of what caregiving entails. As expectations shift, relational responsiveness may be displaced by procedural adequacy as the prevailing standard.

## Introduction

Throughout the history of medicine, technological innovations have transformed how care is given and received. The stethoscope enabled clinicians to access subtle internal rhythms of the body and helped reconfigure diagnostic authority [42], antiseptic techniques fundamentally reduced surgical infection risks [32], and electronic health records standardized documentation and information handling in ways that also reshape the dynamics of clinical encounters [45]. Each innovation not only changed how patients were understood and treated, but also reconfigured aspects of clinical relationships. The current expansion of medical AI raises ethical questions that go beyond efficiency and accuracy. In particular, a subset of patient-facing systems is increasingly presented as able to take over parts of communicative and supportive work (e.g., explaining, reassuring, triaging, or ‘checking in’). In light of these developments, it is important to ask what, if anything, is lost when care-adjacent interactions are automated or even substituted (rather than used to support traditional human caregiving)?

Before evaluating what may be lost in this transition, it is essential to clarify what care itself entails. As a working characterization, I use ‘care’ as a cluster concept that captures dimensions of clinical practice that go beyond technically correct service delivery. Following Tronto’s [53] practice-oriented definition of care as the broad set of activities through which we ‘maintain, continue, and repair our world’ (a definition Tronto attributes to joint work with Fisher), I propose the following three elements as a minimal working characterization of care (i.e., an analytical synthesis rather than a canonical definition): (1) context-sensitive responsiveness to patients’ needs as they arise over time, (2) relational uptake, i.e., attending patients as particular persons rather than merely as a case, and (3) answerability in that someone can be held responsible for how patients’ needs are interpreted, prioritized, and met. Many episodes of care also involve embodied and affective dimensions (e.g., presence, tone, gesture, and sometimes touch). Frankfurt [17] offers a further layer of conceptual depth to this understanding of care, arguing that to care about something is not merely to value it, but to become volitionally bound to it. Caring, for Frankfurt, involves what he calls ‘volitional necessity’—a condition in which one’s will is no longer uncommitted, but is constrained by a deep identification with what matters. This form of necessity does not derive from external moral rules, but from the agent’s inner structure of concern. To genuinely care, then, is to find oneself unable to remain indifferent; the cared-about becomes integral to one’s sense of self. Although Frankfurt focuses on caring *about* rather than caring *for*, his account of volitional necessity helps clarify why caregiving, too, cannot be reduced to behavioral performance.

Following care-ethical discussions [8], one can distinguish ‘caring about’ (i.e., a general orientation of concern) from ‘caring for’ (i.e., a concrete, relational practice of attending and responding to a particular person’s needs). *Caring about* can be expressed broadly and at a distance, whereas *caring for* is typically structured by roles, responsibilities, and ongoing responsiveness, involving questions of answerability and trust. Patient-facing AI can be designed to express forms of concern, and to deliver supportive outputs, but the ethical question in clinical settings is whether such systems can legitimately substitute for the humanly answerable practice of caring for. Since, when we truly care for others, we do so not simply by acting on external obligation, but through a stance of moral responsiveness that is internally anchored. Thus, caregiving is not merely a task, it is a moral stance rooted in the kind of selfhood that binds someone to the suffering of others in a way that cannot be externally imposed. This understanding of care serves as the normative baseline against which the promises and risks of medical AI-driven care will be evaluated.

Now, medical AI covers many heterogeneous systems. My focus here is therefore narrower, as I concentrate on patient-facing AI systems that are designed to engage in care-adjacent interaction in clinical or quasi-clinical contexts (e.g., conversational symptom-triage tools, counseling/support chat bots, and interactive systems presented as companions or communicative intermediaries in care settings). Such technologies make the question of care salient because they are often marketed and adopted as ways to scale (and in some contexts partially substitute for) communicative and relational work traditionally performed within clinical care [36, 37, 46]. By contrast, I largely bracket AI systems used for imaging classification or background administrative optimization, since their ethical issues are real but different from the question of substituting for caregiving roles. This yields two questions that are often run together: (1) In what sense, if any, can patient-facing clinical AI be said to participate in care? (2) Even if such systems can produce interactions that patients experience as supportive, under what conditions (if any) should they substitute for human caregiving roles rather than merely assist clinicians? The first question is partly theoretical, the second is mainly normative. As such, I keep these questions distinct while showing how they bear on each other.

It is important to note that patient-facing clinical AI can be embedded in different professional roles. These include physicians (e.g., triage and treatment decisions), nurses and allied health professionals (e.g., ongoing monitoring, education, discharge planning, and supportive communication), and support workers who provide daily assistance. These roles involve different forms of proximity, continuity, and dependence, and thus different modes of relationship with patients. In what follows, I use ‘clinician/caregiver’ as a role-term for whichever professional (or care team) is answerable for the patient-facing interaction in context. While many AI applications remain focused on clinical decision support and administrative optimization, an increasing subset is designed for patient interaction and is sometimes adopted as a way to scale communication and support. Such systems are positioned not just as a supplement to human care, but as a potential substitute for the relational and affective dimensions traditionally integral to caregiving [36, 37]. For example, socially assistive and companion robots in elder care and adjacent care contexts are often explicitly designed to provide not only task assistance but also forms of social-emotional engagement through conversation, expressive behavior, and affective signaling [1, 2]. I discuss robotic platforms only as one instantiation of patient-facing care-adjacent systems. The argument is not about robots as such but about the substitution of relational work by patient-facing clinical AI more generally. More broadly, a subset of patient-facing AI systems combines speech-based interaction, expressive interfaces, and—in embodied robotic platforms—advances in safer, more ‘skin-like’ tactile sensing intended to support more natural interaction [58]. These advancements raise an important conceptual concern: Could medical AI convincingly replicate genuine relational care? Researchers are already investigating whether robots can exhibit compassion and whether patients might come to trust AI-driven care [31]. These inquiries, however, often fall short of deeper conceptual reflection, rarely addressing the fundamental nature of caregiving itself. Patient-facing medical AI can contribute to healthcare delivery and may even produce interactions that patients experience as attentive or comforting. My claim, however, is that when such systems are deployed as substitutes for caregiving relationships, they cannot occupy the role that clinical practice and care ethics treat as central: being the answerable party who can be held responsible for interpreting vulnerability in context and for revising one’s response over time. This matters not only for authenticity but also for governance and institutional design. In clinical pathways where patient communication and support are routed through AI interfaces, substitution risks shifting the standard of good care from relational responsiveness toward procedural adequacy, thereby gradually reshaping what caregivers, patients, and institutions take care to require.

An inherent ontological limitation of medical AI gives rise to a more fundamental conceptual question: Can patient-facing clinical AI be regarded as an artificial moral agent capable of expanding the traditionally dyadic patient-physician relationship? Some have answered affirmatively, suggesting that machines equipped with complex reasoning, emotional simulation, and context-sensitive responsiveness might eventually satisfy the criteria for moral agency [33]. Yet this perspective overlooks crucial dimensions of authentic moral agency, particularly embodied experience and relational accountability. Baker [3] describes the metaphysical foundations for moral agency as requiring a ‘robust first-person perspective’, the capacity to see oneself as oneself. Additionally, Sparrow [48] argues that machines cannot replace moral agents in contexts where moral authority and responsibility are essential. I take this as an important metaethical backdrop. My aim here, however, is not simply to restate that conclusion. I focus on the care-specific problem regarding how patient-facing clinical AI can produce care-like performances while leaving responsibility structurally ambiguous, and how routine reliance on such performances can contribute to institutional norm shifts that redefine care in terms of procedural adequacy rather than relational responsiveness.

In what follows, I combine empirical evidence with conceptual analysis, exploring the embodied nature of caregiving as a moral practice. Section two examines the physiological and psychological significance of human touch in clinical contexts. Section three addresses the ontological and epistemic limitations of medical AI regarding trust and relational accountability. Section four engages virtue theory, care ethics and phenomenology to argue that genuine caregiving requires embodied moral agency. Section five questions emerging hybrid models of AI-human caregiving and ethics-by-design approaches, highlighting that such approaches risk fragmenting the moral coherence of care. The practical upshot is conditional. Where medical AI is used to augment clinical work while preserving human answerability for interpretive and relational judgments, it may be ethically defensible and clinically valuable. However, where it is deployed as a substitute for relational caregiving—especially in contexts of vulnerability, dependence, and communicative ambiguity—it risks undermining answerability and normalizing a thinner standard of care. The central ethical question is not whether AI can mimic the surface of care, but what healthcare institutions authorize when such mimicry is treated as sufficient.

## The physiological and psychological significance of human touch

Human touch is deeply embedded in physiological processes and psychological well-being; as such, it is much more than mere tactile sensation. I use touch as an illustrative case because it makes vivid how clinical caregiving often operates through embodied, permission-sensitive, and context-dependent forms of responsiveness. The point is not that care always requires touch, nor that mediated or non-human touch can never be beneficial. Rather, touch highlights how caregiving is not exhausted by outwardly similar behaviors. It is embedded in relationships of trust, boundaries, and accountability that unfold over time and that shape what a gesture means in context.

Professional touch in healthcare is not a single type of act, nor is it uniformly experienced as caring. It ranges from diagnostically and therapeutically required contact (e.g., palpation, positioning, mobilizing) to explicitly supportive gestures (e.g., handholding), with many hybrid forms in between. A key feature is that touch is ethically ambivalent; depending on consent, timing, history, and communicative framing, the same bodily contact may be experienced as reassurance and recognition, as merely procedural, or as intrusive and disempowering. Recent work on the ethics and skills of professional touch emphasizes that competent care involves not only technical propriety but also boundary-awareness, permission-sensitivity, and responsiveness to how touch is received in context [28]. Buono et al.’s [5] systematic review of 36 empirical studies argues that the familiar distinction between ‘instrumental’ and ‘expressive’ touch can be misleading, since communicative and affective meaning can arise even in ostensibly procedural touch, and because clinical touch often operates on a fragile boundary between ‘reassuring presence’ and ‘control’. The review also highlights that gender, cultural background, and individual preferences shape both the use and perception of touch, and it frames touch-mediated communication as a co-produced practice grounded in bodily and contextual mutual attunement.

The empirical studies discussed in what follows support two claims. First, that tactile stimulation can have measurable physiological and psychological effects. And second, that these effects are often mediated by how touch is interpreted (e.g., whether it is experienced as supportive or intrusive, and whether it occurs within a relationship of trust). The empirical findings do not, by themselves, establish that only human touch can ever be beneficial, nor do they directly demonstrate ‘moral intentionality’ in touch. The normative question I pursue is instead what is at stake when clinical care is reorganized such that care-like signals that frequently involve touch are produced by AI systems while answerability and relational responsibility are thinned or displaced.

Empirical evidence suggests that, in many clinical contexts, consented and trusted touch can play a multifaceted role [9, 15]. At the biological level, human touch triggers neurophysiological pathways critical for regulating stress and promoting homeostasis. Gentle touch activates C-tactile afferents, specialized nerve fibers linked to emotional and affiliative touch, leading to activation of brain regions such as the insular cortex and the amygdala. This neurobiological cascade results in lowered cortisol levels, reduced heart rate, improved heart rate variability, and decreased blood pressure, offering measurable benefits for cardiovascular and immune function [9]. Although recent empirical research has renewed interest in the role of human touch, its importance has long been recognized. More than a decade ago, Field [15] reviewed over 100 studies showing that massage therapy, hand-holding, and other forms of therapeutic touch consistently reduced cortisol levels, heart rate, and blood pressure, while also increasing serotonin and natural killer cell activity. One study included in her review showed that children with autism experienced an 11% reduction in inattentiveness after receiving 15 min of parent-delivered massage daily for one month. Another experiment revealed that touch as a means of communication could convey emotions with an accuracy ranging from 48% to 83%, demonstrating its crucial role as a nonverbal signal. Additionally, positive shifts in frontal EEG patterns were observed alongside decreased depression and enhanced immune function following moderate-pressure massage. Eckstein et al. [9] similarly synthesized over 150 studies showing that touch interventions reliably dampened psychobiological stress responses across contexts, including intensive care units and oncology wards. Their review highlights experimental evidence that hand-holding during pain anticipation reduced unpleasantness, bodily arousal, and neural threat response with significant effects. Furthermore, participants evaluated hand-holding with a close partner as more relaxing than holding a rubber hand, illustrating the relational specificity of human touch. Psychologically, human touch functions as an embodied signal of comfort, care, and relational presence. As reported in the aforementioned review, even minimal physical contact can significantly reduce feelings of loneliness, anxiety, and emotional distress, particularly in vulnerable populations. These effects are, however, not exhausted by sensory stimulation alone. In several studies, outcomes depend on how touch is appraised in context (e.g., whether it is experienced as supportive or intrusive), suggesting that tactile input is interpreted through relational and normative expectations. Importantly, in several experiments the perceived source of touch (e.g., a close partner versus an artificial proxy) modulates comfort and threat responses, indicating that tactile stimulation is filtered through relational meaning. For example, participants rated human touch as significantly more comforting than robotic touch, even when the tactile stimulation was physically identical [9]. Attempts to replicate human touch through robotic systems have yielded mixed and often underwhelming results. Eckstein et al. reported that in an experiment using a Nao robot to deliver shoulder touches during a stressful video, participants who received robotic touch exhibited lower heart rates but no significant changes in cortisol, skin conductance, or subjective emotional distress compared to controls. Moreover, up to 36% of participants expressed discomfort or preference for no robotic touch, raising concerns about its acceptability. Although this is a robotic touch intervention rather than an AI-only clinical system, I discuss it here because it illustrates a broader issue about technologically mediated touch. Even when tactile stimulation can be delivered, its meaning in clinical care depends on context, consent, and accountable relational judgment. As such, these findings underscore a distinction between delivering mechanical stimulation and delivering touch that is reliably experienced as supportive. Whether touch is welcomed, comforting, or threatening depends on context, consent, professional norms, and the relationship in which it occurs—features that current robotic systems only partially capture, and that, in clinical practice, are ordinarily secured by human judgment and accountability. Accordingly, I treat these studies as evidence about the limits of technological proxies for touch, not as direct evidence about the performance of medical AI in general.

The relational value of human touch also manifests in patients’ attitudes toward AI-mediated care. In an experimental study, Esmaeilzadeh et al. [13] randomized patients into three scenarios: traditional in-person care, AI-assisted care with physician oversight, and AI-only care. Their results showed that patients in the AI-only group reported significantly lower trust, lower satisfaction, and decreased willingness to engage in future care compared to the human and AI-assisted groups. Crucially, the absence of direct human interaction was not merely viewed as a technical inconvenience but as a loss of empathy, accountability, and relational safety. Even when presented with medical AI systems capable of accurate diagnostics, patients preferred models retaining human oversight and physical presence. These preferences align with Field’s [15] broader observation that touch-mediated presence fosters a sense of *being cared for*; an emotional state unlikely to arise from medical AI interfaces alone.

Taken together, the empirical literature supports the physiological and psychological significance of touch in clinical contexts and suggests that its effects are often mediated by context, consent, and interpretation. Touch is therefore not merely a sensory input or procedural adjunct but it can convey relational meanings (e.g., comfort, recognition, reassurance but also intrusion) depending on how it is situated within a relationship and a professional setting. Current technological proxies may deliver tactile stimulation, but they do not reliably reproduce the relational conditions under which touch is experienced as supportive in clinical care. So, if caregiving is partly constituted by answerable, relational responsiveness, what is at stake when care-adjacent interactions are replaced by care-like simulations? For example, consider a patient that has been discharged and receives automated portal ‘check-ins’ from a conversational system that asks about pain, fever, adherence etc., then responds with reassurance, standardized advice, or, as the case may be, escalation prompts. Such a system can mimic the cadence of human follow-ups and may even catch red flags, yet the interaction is structured around scripted thresholds and generic reassurance rather than an accountable relationship in which a medical professional can interpret ambiguity, notice what is not said, and be held responsible for how vulnerability is met.

## Ontological and epistemic limitations of medical AI

To avoid conflating distinct deployments, I distinguish AI-assisted care (i.e., AI supports clinicians who remain answerable for interpretation and communication) from AI-mediated care (i.e., AI structures or filters clinician-patient interaction), and AI-substitutive care (i.e., AI replaces some portion of patient-facing relational work). My central ethical claims target AI-substitutive care and, to a lesser extent, AI-mediated care. I treat AI-assisted care as potentially compatible with regular, traditional care when accountability and interpretive responsibility remain clearly human. That said, patient responses to clinical technologies (such as robot-assisted surgery) also show that perceived non-human agency can elicit fear and anxiety, underscoring how trust and answerability remain central when automation enters clinical practice [35].

Patient-facing medical AI can exhibit ontological and epistemic limitations that are not simply matters of incomplete technology but arise from how contemporary systems are typically built and deployed. Namely, as statistical pattern-recognition systems without first-person experience and without role-based answerability within the clinical relationship. As previously mentioned, care is not merely a task or procedure; it is an embodied, affective, and relational practice grounded in mutual vulnerability. As currently designed, medical AI systems do not possess the kind of embodied, reciprocal presence through which clinicians ordinarily sustain trust, permission, and responsiveness in ongoing care. While medical AI may simulate empathy through verbal cues or affective wording, it does not itself have first-person affective experience. It therefore does not experience empathy, even when it can generate empathic-seeming responses. Pertinent to the kind of empathy that medical AI can simulate, Thompson et al. [51] distinguish between *cognitive* empathy (i.e., the ability to recognize another’s emotional state) and *affective* empathy (i.e., the capacity to emotionally resonate and respond with compassion to another’s emotional state). While medical AI can approximate cognitive empathy (recognizing or classifying emotional cues), it does not thereby acquire affective empathy (emotional resonance that can ground compassion). At most, it can produce outputs that mimic such resonance. Farhud and Zokaei [14] echo this observation in the context of patient-facing, care-adjacent systems, arguing that while such systems may simulate empathic cues, they lack the biological and conscious substrates associated with experienced empathy. They highlight clinical contexts such as obstetrics and pediatrics, where the moral presence of a caregiver, not simply clinical competence, is critical to reducing fear, building trust, and conveying safety. In these settings, the absence of embodied, compassionate presence is not a minor omission. In such settings, the absence of embodied, compassionate presence can be ethically salient, particularly when patient-facing systems are deployed as substitutes rather than supports. Patient focus-group work suggests that trust in AI-mediated care is closely tied to whether a visibly answerable human clinician remains responsible for oversight, whether patients can opt out, and whether recommendations can be contested or corrected [41]. These findings do not establish a uniform phenomenology of ‘alienation’, but they do support a narrower point that aligns with my argument. When AI mediation obscures who is responsible, it can weaken relational safety in ways that matter for caregiving. Patients, particularly children and adults in vulnerable psychological states, may experience AI-mediated care as cold, impersonal, or even alienating. Efforts to humanize medical AI robotics through anthropomorphic design or affective computing risk creating a misleading veneer of simulated empathy. Such simulations may satisfy surface-level expectations, but they lack the existential co-presence and moral accountability that define human caregiving. Stark [50] cautions that efforts to replicate human emotional labor with medical AI can oversimplify complex affective states, inviting what might be called an *ontological blurring*: a confusion between authentic relational care and its technological mimicry. Notably, empirical evidence aligns with these ontological concerns. Richardson et al. [41] report that patients explicitly express unease and discomfort when relational care is mediated or substituted by medical AI. Participants described such interactions as lacking interpersonal trust and accountability, feelings that compounded their sense of vulnerability. Even though these findings do not establish that patients experience the absence of embodied relationality in a direct phenomenological sense. But they do show that patient trust in AI-mediated care is strongly tied to whether an answerable human caregiver remains visibly responsible for the interaction and its consequences.

In addition to ontological limitations, patient-facing medical AI used for care-adjacent interaction faces epistemic limitations in its capacity to ‘know’ patients in morally meaningful ways—limitations that become ethically salient when such systems are used to mediate or substitute for communicative and supportive caregiving. This is so because many contemporary medical AI systems derive their outputs from statistical regularities learned from healthcare datasets that abstract away portions of the context in which data are generated and recorded. As El Mir et al. [11] emphasize, what is often ‘really missing’ is precisely this context, and medical AI systems can thereby amplify misleading, decontextualized data. In that sense, the epistemic orientation of such systems is comparatively statistical and decontextualized relative to clinical reasoning. While medical AI excels at pattern recognition across large datasets, it struggles to engage with the narrative and embodied dimensions of patient lives. Medical AI processes patient histories as data points to optimize predictions,it does not encounter patients as narrative, socially embedded agents. This reductionist orientation not only overlooks contextual meaning but also complicates clinical judgment. As Grote and Berens [21] argue, collaboration between clinicians and medical AI can be undermined by mismatched epistemic stances. When clinicians defer to medical AI without fully understanding, and as the case may be, questioning its outputs, diagnostic performance can actually decrease. Under certain deployment conditions, however—especially where AI outputs are treated as authoritative, where time pressure is high, or where institutional incentives discourage contestation—clinicians may defer to recommendations they do not fully understand or critically assess. In that setting, collaboration can drift into epistemic dependency where the clinician’s role becomes more reactive than interpretive. This is not an inevitable feature of medical AI as such, but a risk that depends on how systems are integrated into workflows and how responsibility is allocated.

Consider a common tele-triage workflow in which a patient first interacts with a symptom-checker or triage chat bot that produces a risk score, a ranked list of likely conditions, and a recommended urgency category. Under time pressure, the clinician may begin the encounter from the system’s structured summary and treat its outputs as the default frame for the consultation (e.g., confirming the checklist rather than eliciting the patient’s narrative). In such a workflow, interpretation becomes downstream of the tool. Instead of exploring context (e.g., atypical symptoms, the patient’s coping capacity, or reasons for delay in seeking care), the clinician primarily reacts to the system’s thresholds. The result is not necessarily worse prediction in every case, but a shift in clinical attention—from contextual sense-making toward procedural confirmation—risking epistemic dependency.

These epistemic limitations of medical AI risk reducing care to transactional data processing, neglecting the relational and affective knowledge crucial for genuine caregiving. Along those lines, Sagona et al. [43] highlight that trust in medical AI is significantly influenced by perceptions of transparency and accountability. Patients reported heightened anxiety and reluctance to engage with medical AI when they felt the medical AI’s recommendations were opaque or unexplainable. Critically, marginalized patients expressed even greater skepticism, fearing that medical AI could amplify biases or overlook context-specific needs. This reinforces the concern that medical AI’s epistemic stance, however accurate computationally, cannot substitute for the narrative, interpretative knowing foundational to relational care. The epistemic gap is further exacerbated by the opacity of many medical AI systems. Wachter et al. [57] warn that medical AI outputs often emerge from complex, non-transparent algorithms (the infamous ‘black box’ problem), making it difficult for clinicians and patients alike to interpret and challenge recommendations. Such opacity undermines shared decision-making and accountability as cornerstones of relational care. If neither patient nor clinician can trace the rationale behind a clinical recommendation, the relational trust foundational to caregiving is weakened. Likewise, Hatherley and Sparrow [24] argue that prioritizing accuracy over interpretability in medical AI risks diminishing trust and harming patient outcomes,a trade-off particularly dangerous in morally sensitive domains like end-of-life care. Vaassen [55] makes the autonomy connection explicit emphasizing that opacity matters not only because it limits interpretability, but because opaque systems can acquire de facto practical authority, constraining people’s capacity for self-governance. In clinical contexts where recommendations can become defaults and where contesting them may be difficult opacity can translate into an autonomy problem. Under certain deployment conditions, reliance on medical AI recommendations can undermine autonomy even when outputs are statistically strong. For example, when recommendations are treated as authoritative defaults, when contesting them is practically difficult, or when patients and clinicians cannot understand the grounds of a decision well enough to deliberate about alternatives. The autonomy concern here is therefore conditional, arising from how authority, explanation, and contestability are structured around medical AI outputs, not from predictive accuracy alone.

In what follows, I use several related terms that should not be run together. The distinctions I draw are based on general philosophical treatments of these concepts, which I apply here to the clinical setting. ‘Epistemic authority’ concerns who is taken to be a reliable source of knowledge or recommendation in a clinical workflow [19]. ‘Responsibility’ concerns who is expected to act (and to prevent or repair harm) in light of that recommendation [16]. ‘Accountability’ concerns who can be called upon to justify decisions, and who bears institutional or professional consequences when things go wrong [4]. ‘Moral agency’ concerns the capacity to be an appropriate subject of such demands for justification (including acknowledgment of reasons, responsiveness to criticism, and the possibility of blame or remorse) [16]. Finally, ‘trust’ in clinical contexts is not just predictive reliability, but it includes the expectation that the trusted party is answerable and will respond appropriately to one’s vulnerability over time [22]. Now, when AI systems become focal points of epistemic authority, they can reshape how responsibility and accountability are distributed. The ethical concern is not that AI ‘has’ responsibility, but that its integration can obscure the human answerability that clinical care requires.

These ontological and epistemic concerns also have an experiential correlate for patients in clinical pathways where patient-facing AI substitutes for (or substantially reduces) human interaction. When routine check-ins and narrative elicitation are routed through AI interfaces, opportunities for meaningful human contact can shrink, which may exacerbate feelings of isolation. An issue already salient in healthcare systems strained by pandemic conditions and staffing pressures [30]. The claim here is not about consumer ‘social AI’ outside healthcare, but about institutional uses of patient-facing AI within clinical care, where conversational interfaces or companion-like systems are introduced as replacements for bedside interaction. Such systems may offer momentary engagement, but they do not supply the reciprocal relationship and answerability that relational care presupposes. They can, therefore, mask, rather than remedy, relational deprivation.

The proliferation of medical AI risks creating a healthcare environment where relational interaction is treated as an optional ‘soft skill’. Patients may comply with AI-driven instructions, but they may nonetheless be less likely to experience themselves as cared for in the ‘morally thick’ sense associated with relational care and responsiveness [25, 52]. This concern aligns with patient focus-group findings emphasizing the importance of human oversight, answerability, and choice in AI-mediated healthcare [41]. Richardson et al. [41] emphasize related concerns about relational safety. In particular, the importance of physician-led oversight, the ability to opt out, and the ability to dispute or correct algorithmic recommendations. In patient-facing conversational deployments, these conditions matter because candid disclosure depends on perceived answerability. When AI-mediated interaction obscures who is responsible, it can undermine trust in ways that may inhibit what patients are willing to share. This reluctance underscores a key insight: empathy, trust, and relational safety cannot be abstracted from human presence. Still, one might argue that simulated embodied relational care (whether delivered through patient-facing AI-enabled robotic or haptic systems or through simple mechanical devices) is preferable to its absence altogether. A compelling example of simulated touch emerged during the COVID-19 pandemic, when interventions were developed for patients isolated in intensive care units. Although this intervention is not an AI system, I discuss it as a mechanistic proxy that helps separate the biological effects of tactile stimulation from the relational and accountability dimensions of clinical touch. Karaman et al. [27] describe the use of a simple yet evocative device known as the ‘love glove’: a latex glove filled with warm water and placed in a patient’s hand to replicate the sensation of human touch (a technique long familiar in veterinary medicine). Their study reported measurable physiological improvements, including increased oxygen saturation and reduced systolic and diastolic blood pressure, following the use of the glove. While such outcomes underscore the biological responsiveness to tactile stimulation, the intervention remains fundamentally a simulation. It provides mechanical warmth and pressure but lacks the relational presence of a human caregiver. Its effectiveness illustrates that certain biological benefits of touch can be mechanistically replicated, but it simultaneously affirms the ontological and epistemic limitations of substituting embodied, relational care with technological proxies. Notably, the study did not compare the glove’s effects directly with actual human touch. However, such a comparison would be essential to determine whether the simulated intervention can meaningfully approach the therapeutic value of actual human touch. Needless to say, this was not feasible during the pandemic, when minimizing direct physical contact was a necessary precaution.

The ontological and epistemic limitations discussed above suggest that medical AI does not straightforwardly occupy the role of a caregiver within a relational practice of care—especially when deployed as a substitute rather than as clinician support. Medical AI’s mode of understanding is grounded in abstract, decontextualized data analysis rather than in experiential knowledge. As a result, medical AI is not well suited to interpret patient experiences in the morally meaningful, context-sensitive way that clinical caregiving ordinarily requires. Nor does it, by itself, secure the transparency and accountability that underpin shared decision-making in patient-centered care. If medical AI is unable to ‘care’ in the full moral sense, responsibility falls to those who design, implement, and regulate its use in healthcare to ensure that its application does not compromise the moral foundations of caregiving. As the foregoing patient-facing examples illustrate, the central concern is no longer technical capability alone, but how particular deployments of conversational triage, automated follow-up, and AI-mediated encounter-structuring can reconfigure the moral ecology of caregiving. At the core of this moral ecology lies the clinician’s moral agency regarding AI-mediated decision-making. As Grote and Berens [20] argue, the integration of medical AI may incentivize clinicians to rely on such systems not because of epistemic confidence, but out of a desire to avoid legal liability. This form of defensive medicine risks turning caregiving into a procedural exercise, where moral judgment and personal accountability (as hallmarks of clinicians’ moral agency) are challenged by deference to AI authority. Rather than enhancing care, then, such dynamics may erode clinicians’ moral agency, undermining the kind of trustful patient-doctor relationship that is foundational to healthcare. Recognizing these risks, I now turn to the moral foundations of care itself.

## The ethics of care beyond simulation

Virtue theory provides a normative framework for understanding why relational care neither can nor should be reduced to codifiable tasks. Rather than focusing on rules or outcomes, virtue theory emphasizes the cultivation of moral character and practical wisdom (*phronesis*) through embodied experience [26]. Applied to caregiving, this tradition foregrounds virtues central to healthcare (e.g., compassion, patience, attentiveness, veracity, and trustworthiness), not as mechanical actions but as expressions of an integrated moral life shaped by mutual responsiveness and accountability. As such, virtue theory anticipates the ethical shortcomings of attempts to simulate care through medical AI. As an illustrative case, Sharkey and Sharkey [46] examine the use of care robots in elderly care settings and argue that while these machines may mimic the outward behaviors of caring, they lack both the emotional attunement and relational understanding that give such behaviors their moral footing. Their argument underscores a key insight from virtue theory: caregiving is not merely what one does, but *how* and *why* one does it. Patient-facing AI systems can imitate surface markers associated with care (e.g., empathic phrasing, conversational mirroring, supportive prompts). However, such performances do not, by themselves, supply what is ethically central in clinical care; namely, answerability for interpreting vulnerability in context and for being held responsible when interpretation and response go wrong. This emphasis on responsibility and responsiveness is central to care ethics, where ‘taking responsibility’ is treated as a core moral moment of care and moral failure is often understood as a failure of responsibility and responsiveness within relations of vulnerability [29, 52].

While virtue theory highlights the moral character and cultivated dispositions of the caregiver, care ethics complements this perspective by drawing attention to the contextual nature of caregiving. More specifically, virtue ethics helps explain how caregiving involves practical wisdom and stable dispositions such as compassion, patience, and attentiveness. Care ethics, on the other hand, emphasizes that these dispositions matter because caregiving is a relational practice shaped by vulnerability and dependency, and because it involves responsibilities that must remain answerable to the person cared for. I draw on virtue ethics and care ethics in a complementary way to capture both the moral capacities of caregivers and the relational conditions under which those capacities are exercised. On this view, care is not simply the expression of virtuous traits, but a morally charged practice shaped by concrete interactions, power asymmetries, and ongoing responsibilities to vulnerable others. Foregrounding qualities such as attentiveness, responsibility, responsiveness, competence, and solidarity [52], which emerge not in isolation but through situated, often asymmetric relationships. Care ethics thus helps reveal what is lost when caregiving is reduced to technical function or simulated empathy,namely, the moral substance of care as a lived, intersubjective practice. Kittay [29] enriches this account by centering care on human dependency and interdependence, arguing that dependency is not a moral shortcoming but a fundamental and enduring condition of human life. Caregiving must recognize and honor this dependency, not obscure it under false pretenses of autonomy or independence. On Kittay’s view, caregiving is often structured by relations of dependency and vulnerability. The moral demand, however, is not merely that caregivers assume responsibility over another person. Rather, ethically adequate care requires a relational commitment to the particular person’s well-being that is responsive to their expressed needs, preferences, and voice. This avoids paternalistic substitution of the caregiver’s judgment for the cared-for person’s agency wherever possible. Disability-rights and independent-living scholarship has long warned that ‘care’ can be associated with paternalism and loss of control when disabled people are positioned primarily as passive recipients rather than participants with agency [10, 38]. Dependency relations require ongoing negotiation of autonomy, support, and accountability within the relationship. This is the sense in which ‘answerability’ matters for the here voiced concerns regarding patient-facing AI. Not as unilateral authority, but as the obligation to remain responsive and accountable within a relationship that recognizes the cared-for person as a participant rather than a passive object of care. Medical AI, however, cannot meaningfully engage in these morally loaded relationships because it does not itself stand in relations of dependency and vulnerability in the care-ethical sense (as a being whose needs can call for care), and it cannot occupy the role of an answerable moral agent within such relations [29, 48, 52].

More recently, this understanding of care has been refined by shifting the normative emphasis from dependency to vulnerability. Engster [12] argues that vulnerability is a more universally applicable foundation for care ethics, highlighting that all of us are perpetually vulnerable in different ways. Such emphasis on vulnerability as a basic human condition strengthens the moral justification for care by underscoring the continuous need for relational support, not only in conditions of visible dependency but throughout the course of people’s entire life. Van Dijke et al. [56] contribute a complementary perspective to the notion of perpetual vulnerability by emphasizing that care ethics demands a nuanced understanding of empathy. Not, however, understood as a fixed trait, but as a relational and context-sensitive mode of moral engagement. They highlight the epistemic and normative limitations of empathy, including risks of projection, misrecognition, and power asymmetries. These concerns are particularly salient in the context of medical AI, where affective simulations may reproduce the surface features of empathy without embodying its moral footing. Care ethics sheds light on how such simulations bypass the embodied and morally grounded responsiveness that caregiving demands, particularly its sensitivity to vulnerability and accountability.

Along these lines, Montemayor et al. [37] argue that empathy represents a principled limitation for medical AI. While medical AI may simulate cognitive empathy (e.g., inferring emotional states based on cues) it lacks the emotional resonance required for authentic moral concern. This absence is not a mere technological shortcoming,it introduces the risk of creating systems that resemble human psychopaths: skilled at reading emotions but incapable of caring. To illustrate the potential danger, Montemayor et al. introduce the term ‘empathy*’ to describe affective mimicry that elicits emotional responses without moral substance. These simulations, they argue, may not only be inadequate but manipulative, misleading patients into projecting trust onto systems that cannot reciprocate relational presence. Montemayor et al.’s concern is further supported by Fuchs’ [18] phenomenological view of relational understanding. For Fuchs, genuine empathy arises not from inferential accuracy and behavioral mimicry, but from embodied co-presence as a mutual attunement grounded in our shared vulnerability. AI, Fuchs tells us, cannot enter what he calls the space of ‘conviviality’ (i.e., a space of shared embodied, affective, and intersubjective experience), as it lacks both subjective embodiment and affective intentionality. At best, AI evokes what Fuchs calls an ‘as-if consciousness’, prompting users to project emotional presence onto a system that, albeit, has no subjectivity. Coeckelbergh’s work helps to situate this phenomenon without treating it as merely a psychological error.

Coeckelbergh [6] argues that, in practice, humans ascribe agency and even forms of responsibility not by inspecting inner states but on the basis of how an entity appears and performs in interaction,he calls this ‘virtual moral agency’ and ‘virtual moral responsibility’. On this view, it is unsurprising that empathic performances by patient-facing AI can invite patients to relate to a system as if it were a morally responsive partner. What matters morally is not only what the system ‘is’, but how it is encountered and how it reorganizes moral practices [7]. At the same time, precisely because these ascriptions are grounded in appearance rather than genuine participation in accountable moral relations, they can obscure where answerability lies in clinical care, making it easier to misdirect trust toward systems that cannot reciprocate responsibility. Such projection can create an ontological confusion: an illusion of moral responsiveness where none exists. In the context of healthcare, this illusion risks displacing genuine caregiving with affective performances that conceal the absence of genuine moral accountability. The more convincingly medical AI simulates care, the more it threatens to replace Fuchs’ space of conviviality with scripted performances that mimic moral concern without participating in it. These conceptual concerns find empirical confirmation in recent work by Seitz [44], who shows that when healthcare chatbots simulate empathy (whether affective, cognitive, or behavioral) they are often perceived as warmer, but at the same time less authentic. This ‘authenticity paradox’ undermines trust and willingness to engage, ultimately eroding the very relational benefits empathy is meant to provide. Notably, this effect appears specific to human-AI interactions; in comparable human–human settings, expressions of empathy do not elicit the same distrust.

The ontological confusion evoked by simulated empathy may be more dystopian still. We are not only faced with the absence of moral agency in medical AI, but with the risk that the very meaning of caregiving may be gradually redefined. As medical AI becomes more fully integrated into healthcare, it may simultaneously reflect and reinforce a moral ecology already moving toward efficiency, affective simulation, and procedural regularity. In this sense, medical AI can be understood both as a symptom of this orientation and as a factor that further entrenches it by making care-like simulations appear sufficient. In this way, medical AI does not simply fall short of existing moral standards of care; it subtly redefines them, normalizing relational minimalism and algorithmic proxies as the new good enough. Sharkey and Sharkey [46] articulate a similar concern, warning that ‘carebots’ risk creating illusions of care that may satisfy superficial needs while undermining its relational basis. The promise of robotic companionship, they tell us, threatens to normalize substitution rather than supplementing human presence, ultimately reducing rather than enriching the moral ecology of care. This shift introduces a moral-phenomenological transformation where the nature of care itself changes under medical AI’s presence. Patients and clinicians alike may come to see care as a service provided rather than a relationship entered into—a set of outcomes to optimize rather than a vulnerability to accompany. The worry is not only in failing to replicate care, but in redefining it to fit what medical AI can provide. Over time, such a redefinition risks deskilling and inhibited upskilling among caregivers by shifting clinical work toward deference to automated outputs and scripts, thereby reducing opportunities to practice and develop core competencies, including clinical judgment and clinician-patient communication [39]. As Held [25] emphasizes care is not a ‘soft’ virtue, but a foundational moral orientation essential for personal, social, and political life. When genuine care is permeated by technical substitutes, the moral ecology that sustains human flourishing begins to dissolve. Held challenges the implicit devaluation of care in systems oriented toward autonomy and efficiency alone. Integrating medical AI into caregiving without centering care’s moral footing perpetuates the very injustices that care ethics aims to address.

In sum, virtue ethics, care ethics, phenomenology, and empirical research converge on a shared moral insight: caregiving is a relational, embodied, and morally accountable practice that is irreducible to rules, functions, and simulations. While medical AI may augment clinical work, patient-facing systems do not thereby cross into genuine care in the relevant sense—unless a human caregiver remains answerable for interpretation, responsiveness, and the repair of breakdowns in the relationship. More than a technical limitation, its widespread integration risks transforming caregiving into a managerial process. Because medical AI lacks embodied vulnerability and reciprocal participation, it cannot by itself supply the relational answerability that characterizes genuine caregiving in clinical contexts. Taking the nature of care seriously, then, demands more than cautious technological design; it calls for a defense of the irreplaceable role of moral agency. Any integration of medical AI must, therefore, be evaluated not only in terms of clinical efficiency, but also with respect to its effects on the moral ecology of care.

## Ethical challenges of hybrid AI-human care models

Hybrid care models, in which patient-facing medical AI supports rather than supplants human caregivers, are frequently proposed as a response to the ethical unease surrounding the technological displacement of human care. At a first approximation, combining computational efficiency with human empathy appears promising. Yet a closer look reveals ethical concerns that extend beyond technical design, including the fragmentation of care, obscured accountability, and the erosion of relational trust. Consider a clinic that deploys a conversational triage and follow-up system in its patient portal. Patients describe symptoms in natural language; the system generates a structured summary for clinicians, proposes urgency categories, and sends automated messages such as reassurance, self-care guidance, or reminders to seek urgent care if certain thresholds are crossed. Even though no robot is involved, this is a patient-facing system that can take over parts of communicative and supportive work. The ethical question, then, is whether this workflow preserves clear human answerability for interpretation, deliberation, and repair when misunderstandings occur. Or whether it functions, in practice, as a partial substitute for relational caregiving.

Hybrid care models typically delegate cognitive and procedural tasks to medical AI while reserving emotional labor and moral responsibility for human caregivers. Although this division may appear efficient, it risks fragmenting care into disjointed functions, thereby undermining its holistic and relational character [46]. When AI-based decision-support is treated as authoritative in diagnosis or treatment planning, patients may experience a perceived split between algorithmic authority and human empathy, even though clinicians retain final responsibility. Such division of roles also complicates moral accountability. Mittelstadt et al. [34] argue that the opacity of algorithmic systems makes it difficult for clinicians to fully interpret or challenge AI-generated outputs. In patient-facing contexts, this matters relationally because opacity can erode answerability. Clinicians may be less able to justify decisions to patients or repair trust when recommendations are contested. When responsibility is ambiguously shared between clinicians and medical AI, trust in caregiving relationships becomes precarious. Yet not all scholars regard opacity in medical AI as inherently corrosive to trust and patient autonomy. Prince and Lim [40] argue that even in the presence of algorithmic opacity, autonomy can be preserved, provided that clinicians actively frame medical AI outputs within a relationship of trust and open communication. On their account, the ethical burden shifts from technical transparency to clinicians’ interpretive responsibility, underscoring the critical role of human mediation in sustaining moral accountability. Still, even with human oversight, opaque algorithms introduce new layers of epistemic uncertainty into clinical encounters. When patients suspect that critical decisions are shaped by inscrutable systems, their trust in both caregivers and institutions may erode. And yet, trust remains foundational to the doctor-patient relationship. However, as Sharkey and Sharkey [46] note, this trust may erode when medical AI influences care in ways that are not transparent. Patients may feel alienated, particularly in sensitive contexts like palliative care or mental health settings, when interactions are filtered through medical AI, even when a human caregiver is present. This risk of such dehumanization is not merely speculative. Sharkey and Sharkey point out that when medical AI significantly mediates caregiving, patients may perceive the experience as transactional, feeling more managed than truly cared for. Hybrid models can unintentionally reinforce such perceptions of being managed by using medical AI to structure relational engagement.

The ethical concerns, however, extend beyond interpersonal dynamics to the data infrastructures that underpin hybrid care. If medical AI systems are trained on biased or incomplete datasets, their outputs may perpetuate existing health disparities, even when mediated by human oversight. Mittelstadt et al. [34] emphasize that the presence of a human caregiver does not automatically neutralize the structural biases embedded within medical AI. This does not imply that clinicians are bias-free. Rather, the concern is that AI-mediated workflows can repackage, amplify, or obscure bias while simultaneously diffusing responsibility, making biased outcomes both harder to detect and contest. These biases persist, particularly when medical AI remains opaque in clinical contexts. Responding to such concerns, proponents of hybrid models often turn to ‘ethics-by-design’ as a safeguard, asserting that embedding ethical principles into medical AI development will ensure its responsible integration. However, Mittelstadt et al. argue that this takes an overly abstract and formalistic stance. A similar point is made in the healthcare robotics literature. Stahl and Coeckelbergh [49] argue that addressing ethical issues requires approaches that stay close to contexts of use and innovation practices, emphasizing dialogical and embedded forms of ethical reflection rather than treating ethics as a one-time design-stage compliance exercise. Ethical principles encoded at the design stage may fail to guide real-world decisions, especially under institutional pressures or in complex clinical environments. When ethics is reduced to technical compliance or procedural checklists, it threatens to sideline the context-sensitive dimensions of moral judgment. In so doing, ethics-by-design may displace moral responsibility from caregivers onto developers, effectively passing the buck in ways that obscures accountability in complex situations. By relying on ethics embedded at the technical level, proponents of hybrid care models overlook the need for ongoing moral deliberation at the point of care. The moral complexity of real-world caregiving cannot be fully pre-engineered, as it requires human responsiveness. Ethics-by-design frameworks may provide a foundational structure for a more responsible integration of medical AI, but they are no substitution for the embodied moral reasoning necessary in caregiving.

Additional challenges arise from the evolving nature of adaptive medical AI systems that adapt across time and contexts. Hatherley and Sparrow [23] draw attention to two forms of variability in medical AI that disrupt clinical consistency: ‘diachronic variation’, in which a medical AI’s outputs shift over time as it continues to learn, and ‘synchronic variation’, where identical medical AI models behave differently across sites due to local data and implementation differences. These instabilities complicate clinicians’ ability to anticipate and explain medical AI, making both informed consent less meaningful and introducing equity concerns, as patients may unknowingly receive unequal standards of care. These issues are compounded by a broader systemic tendency to treat medical AI recommendations as authoritative, even when their reliability varies unpredictably. Recognizing this, Solanki et al. [47] call for ethically grounded design frameworks that empower developers to take responsibility throughout the entire medical AI lifecycle (i.e., from data curation to deployment and post-deployment monitoring). However, the authors also acknowledge a key limitation of technical solutions as they cannot substitute for the situated moral judgment required in real-world care. Taking this concern further, Ugar [54] argues that the widespread integration of medical AI may inadvertently displace patients as participants in reason-giving and shared decision-making about their own care. Thus, when decision-making authority is shifted toward opaque medical AI, patients risk being positioned as passive recipients rather than active participants in their care, undermining the very idea of shared decision-making. This not only undermines moral agency but also erodes relational accountability. These theoretical concerns find empirical support in a cross-sectional vignette study by Zondag et al. [59], which shows that patients express diminished trust in physicians who rely on AI-based decision-support systems during high-risk clinical encounters. Patients reported lower confidence not only in the technology, but also in the clinicians themselves, suggesting that AI-mediated care may fracture the moral bonds of trust that define clinical relationships.

## Concluding remarks

In this paper, I have focused on patient-facing clinical AI used for care-adjacent interaction. My central claim is not that such systems cannot be clinically useful, nor that they can never be experienced as supportive. Rather, the argument concerns their role and answerability. When patient-facing AI is deployed in ways that substitute for communicative or supportive work that ordinarily functions within an accountable caregiving relationship, it does not straightforwardly occupy the caregiving role that care ethics and clinical practice treat as morally central. Namely, being the answerable party who can be held responsible for interpreting vulnerability in context, revising responses over time, and repairing breakdowns in trust.

This yields a practical, conditional upshot that mirrors the deployment distinctions developed above. AI-assisted care can be ethically defensible and clinically valuable when clinicians remain clearly answerable for interpretive and relational judgment, including the framing of recommendations, permission-sensitivity, and accountability when things go wrong. By contrast, AI-mediated and especially AI-substitutive care risks thinning or even displacing answerability. It can reorganize care around procedural adequacy and care-like performance while leaving responsibility structurally ambiguous. Over time, this matters not only for individual encounters but for institutional norms. When healthcare pathways come to treat care-like outputs as sufficient, the standard of good care may shift from relational responsiveness toward procedural regularity—thereby reshaping what caregivers, patients, and institutions take care to require.

Sparrow’s [48] broader point that machines cannot replace moral agents provides an important metaethical backdrop for what I have argued. But the care-specific concern emphasized here is how patient-facing clinical AI can generate persuasive care-like performances while obscuring the locus of responsibility in contexts of vulnerability and dependence. Taking the moral ecology of care seriously, therefore, requires more than technical refinement. It requires governance and clinical design choices that preserve human answerability where caregiving is morally indispensable. Treating care-like simulation as, at most, a supplement to—rather than a replacement for—answerable clinical care. One normative implication is that when caregiving is approached as a substitutable service output rather than an answerable moral practice, what is at stake is not only clinical workflow but also how we understand and enact our moral commitments. If, as Frankfurt [17] reminds us, what we care about defines who we are, then how we treat caregiving reflects not just our clinical priorities but also our moral selfhood. To allow caregiving to become an artificial performance is not only to alter what we care *for*, it is to diminish what we care *about*.

## Data Availability

No datasets were generated or analysed during the current study.

## Abbreviations

AI: Artificial Intelligence
COVID: Coronavirus Disease
EEG: Electroencephalogram
